# Antidiabetic Rosiglitazone Reduces Soluble Intercellular Adhesion Molecule-1 Level in Type 2 Diabetic Patients with Coronary Artery Disease

**DOI:** 10.1155/2008/548178

**Published:** 2008-12-18

**Authors:** Guang Wang, Zhe Zhang, Jie Yu, Fuchun Zhang, Liyun He, Jinru Wei, Jieming Mao, Xian Wang

**Affiliations:** ^1^Institute of Vascular Medicine, Peking University Third Hospital, Beijing 100191, China; ^2^Department of Cardiovascular Surgery, Peking University Third Hospital, Beijing 100191, China; ^3^Department of Physiology and Pathophysiology and Key Laboratory of Molecular Cardiovascular Science, Ministry of Education, Peking University Health Science Center, Beijing 100083, China

## Abstract

*Background*. We investigated the level of soluble adhesion molecules in diabetic patients and the effect of the peroxisome proliferator-activated receptor-*γ* (PPAR-*γ*) agonist rosiglitazone on plasma levels of adhesion molecules and an inflammation marker in type 2 diabetic patients with coronary artery disease (CAD) after percutaneous coronary intervention (PCI). *Methods*. A total of 116 diabetic patients with CAD who had undergone PCI were randomized to receive rosiglitazone (4 mg/d) or not for 6 months. Plasma levels of soluble intercellular adhesion molecules (sICAM-1) and P-selectin (sP-selectin) were measured on ELISA. *Results*. After 6-month rosiglitazone treatment, plasma levels of sICAM-1 were lower than baseline and control group levels (370.4 (332.4–421.9) pg/mL versus 423.5 (327.4–500.3) pg/mL and 404.6 (345.2–483.4) pg/mL, *P* < .001). In addition, plasma levels of C-reactive protein were significantly reduced from baseline levels. However, plasma level of sP-selectin was not significantly lowered with rosiglitazone treatment than with control treatment after 6-month follow-up. *Conclusions*. Rosiglitazone reduces chronic inflammatory responses and improves levels of markers of endothelial dysfunction in patients with diabetes and CAD. PPAR-*γ* agonist may have a beneficial effect on the vascular endothelium through its anti-inflammatory mechanism and may be useful as therapy in patients undergoing PCI.

## 1. INTRODUCTION

Chronic subclinical inflammation is increasingly recognized as possibly contributing to the progression of atherosclerosis and acute coronary syndromes. The formation and development of atherosclerotic lesions involve accumulation of monocytes and T lymphocytes [1]. The process of leukocyte adhesion and transendothelial migration is mediated by cellular adhesion molecules, which are expressed on the endothelial surface in response to many atherogenic stimuli [2]. Elevated plasma levels of soluble adhesion molecules suggest a role in plaque instability and predict the development of atherosclerosis and cardiovascular events in patients with coronary artery disease (CAD) [3, 4]. In addition, several prospective studies have demonstrated that plasma level of C-reactive protein (CRP) is an inflammatory marker of cardiovascular disease. Chronic subclinical inflammation is part of the insulin resistance syndrome [5]. Although the exact cause of atherosclerosis is not clear, the improvement of metabolic disorders and chronic inflammation characterized by insulin resistance may significantly decrease the risk of the disease.

Peroxisome proliferator-activated receptors (PPARs) are ligand-activated transcription factors that are a subfamily of the nuclear receptor gene family. PPAR-*γ* plays a central role in metabolism and is highly expressed in endothelial cells, vascular smooth muscle cells, and macrophages [6]. The rosiglitazones are a class of pharmacological compounds with high affinity to PPAR-*γ*. However, PPAR-*γ* agonists reduce plaque inflammation by inhibiting the activation of several proinflammatory genes responsible for plaque stability through decreasing the expression of adhesion molecules [7, 8]. Troglitazone inhibits the interaction between leukocytes and endothelial cells, decreases the expression of intercellular adhesion molecule-1 (ICAM-1) in activated endothelial cells, and reduces monocytes homing to atherosclerotic plaque [9, 10]. Furthermore, our previous study demonstrated that PPAR-*γ* agonists significantly reduce homocysteine-induced reactive oxygen species and secretion of monocyte chemoattractant protein-1 (MCP-1) in monocytes [11]. Recently, we showed plasma levels of MCP-1 significantly decreased with rosiglitazone treatment [12, 13]. However, whether PPAR-*γ* agonists can exert a beneficial effect on soluble adhesion molecule levels to promote adhesion and transendothelial migration of monocytes to endothelium in patients with diabetes and CAD after percutaneous coronary intervention (PCI) is unknown.

We aimed to investigate the level of soluble adhesion molecules in diabetic patients and the effect of rosiglitazone on plasma levels of adhesion molecules and an inflammation marker in type 2 diabetic patients with CAD after PCI.

## 2. METHODS

### 2.1. Subjects

Patients were selected from the cardiovascular internal medicine practice at Peking University Third Hospital between October 2002 and September 2005. We included 116 patients, aged 45 to 79 years old, with a diagnosis of CAD (>50% stenosis seen on angiography) and type 2 diabetes mellitus. Patients with acute myocardial infarction during the preceding 12 weeks, cardiac insufficiency, renal function impairment, liver function impairment, systemic inflammatory disease, infectious disease, cancer, or a serious illness that would affect their participation or patients under insulin treatment were excluded.

### 2.2. Study design

All 116 patients had undergone angiography and percutaneous coronary intervention. The patients were divided into two groups for treatment depending on sequence of patients recruited into the study: control group (56 patients) and rosiglitazone group (60 patients), who received 4 mg rosiglitazone daily for 6 months. One patient in the treatment group withdrew during follow-up because of recruitment into another clinical trial. Blood was sampled before and after angiography for 6 months for analysis of clinical chemistry and inflammatory factors. Plasma was separated and stored at −70°C for further analysis.

All subjects gave their written, informed consent. This study was approved by the Ethics Committee of the Health Science Center, Peking University.

### 2.3. Laboratory measurements

Blood samples were drawn from an antecubital vein in the morning after overnight fasting and collected into vacuum tubes containing EDTA to measure plasma lipid levels. Total cholesterol (TC), high-density lipoprotein cholesterol (HDL-C), low-density lipoprotein cholesterol (LDL-C), and triglyceride (TG) levels were analyzed by colorimetric enzymatic assays with use of Auto-Analyzer (HITACHI-7170). The measurements of fasting plasma glucose, fasting insulin, and hemoglobin A1c were determined at the Central Chemistry Laboratory, Peking University Third Hospital.

Levels of CRP were measured by the use of an enzyme-linked immunosorbent assay (ELISA) kit following the manufacturers' protocols (R&D systems, Minneapolis, Minn, USA). Plasma levels of soluble ICAM-1 (sICAM-1) and P-selectin (sP-selectin) were measured by ELISA (GeneMay, Inc., San Diego, Calif, USA) according to the manufacturer's protocols. The estimate of homeostasis model assessment-insulin resistance (HOMA-IR) was as follows: IR = (fasting insulin (*μ*U/mL) × fasting glucose (mmol/L))/22.5.

### 2.4. Statistical analysis

Differences between groups in levels of glucose, TC, LDL-C, HDL-C were analyzed by Student's *t* test. Values for continuous variables are expressed as means ± SD. Plasma levels of soluble ICAM-1 are given as medians and ranges and were assessed by non-parametric tests (Mann Whitney *U*-test). Proportions were analyzed by use of the chisquare test. A value of *P* < .05 (two-tailed) was considered statistically significant.

## 3. RESULTS

### 3.1. Clinical characteristics of patients

The characteristics of patients are summarized in Table 1. The control and rosiglitazone groups did not differ significantly in baseline demographics; risk factors for atherosclerosis; prevalence of smoking, hypertension, hyperlipidemia, and use of other medications; as well as laboratory values or plasma levels of inflammatory factors. The two groups did not differ in medical therapy during 6-month follow-up other than use of rosiglitazone or in diabetic duration or complications.

### 3.2. Effects of rosiglitazone treatment on metabolic parameters

**Figure 1:**
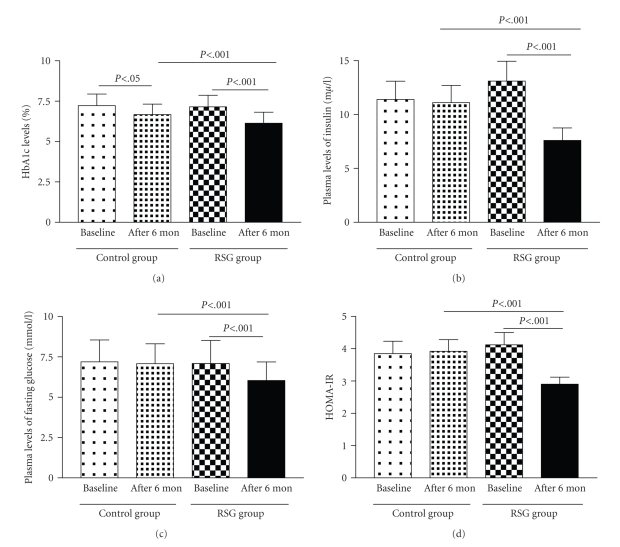
(a) Changes in levels of hemoglobin A1c, (b) fasting plasma insulin, and (c) glucose, as well as (d) HOMA-IR, with 6-month rosiglitazone treatment. Data are means ± SD. Control group, *n* = 56; rosiglitazone (RSG) group, *n* = 60. HbA1c: hemoglobin A1c; HOMA-IR: homeostasis model assessment-insulin resistance.

After 6-month rosiglitazone treatment, the level of hemoglobin A1c was significantly decreased in the rosiglitazone group as compared with baseline and control levels (Figure 1(a); 6.14 ± 0.68% versus 7.15 ± 0.72% and 6.67 ± 0.65%, *P* < .001). Similarly, rosiglitazone significantly decreased the level of fasting plasma insulin and glucose as compared with baseline and control levels (7.60 ± 1.15 mmol/L versus 13.10 ± 1.84 mmol/L and 11.10 ± 1.60 mmol/L, *P* < .001; 6.04 ± 1.15 mmol/L versus 7.10 ± 1.42 mmol/L and 7.08 ± 1.23 mmol/L, *P* < .001; Figures 1(b)-1(c)). HOMA-IR levels were significantly decreased after 6-month treatment (2.9 ± 0.32 versus 4.12 ± 0.38 and 3.92 ± 0.36, *P* < .001, Figure 1(d)), as expected. However, hemoglobin A1c and fasting plasma glucose levels were not significantly different from the control group at 6-month follow-up (Table 1). In addition, plasma levels of TC, HDL-C, LDL-C were significantly decreased with rosiglitazone treatment as compared with baseline levels, with no significant difference between the rosiglitazone and control group at 6-month follow-up (Table 2).

Weight gain can be a major drawback in treatment with a PPAR-*γ* agonist. Body weight was increased but not significantly from the baseline and control levels after 6-month rosiglitazone treatment (Table 2).

### 3.3. Rosiglitazone effect on plasma levels of inflammatory markers sICAM-1, sP-selectin, and CRP

**Figure 2:**
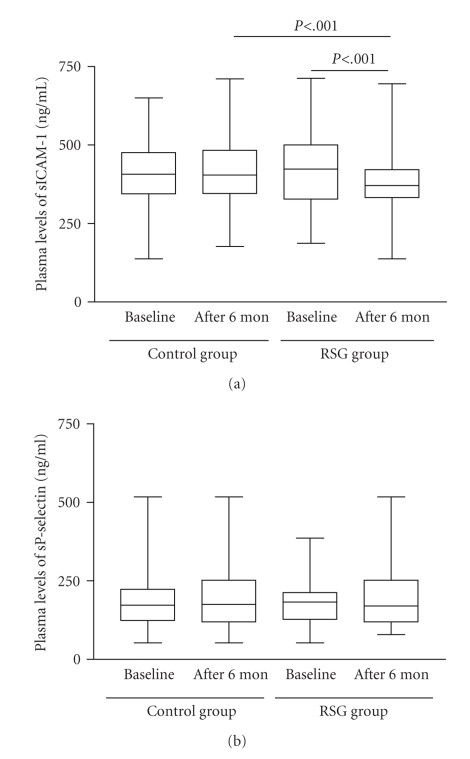
Effect of rosiglitazone treatment on plasma levels of soluble ICAM-1 (sICAM-1) (a) and P-selectin (sP-selectin) (b) in patients with diabetes and coronary artery disease after percutaneous coronary intervention. Data are medians and ranges. Control group, *n* = 56; rosiglitazone (RSG) group, *n* = 60.

Plasma sICAM-1 levels were significantly decreased in the rosiglitazone group compared with baseline and control levels after 6-month treatment (370.4 (332.4 to 421.9) pg/mL versus 423.5 (327.4 to 500.3) pg/mL and 404.6 (345.2 to 483.4) pg/mL, *P* < .001) (Figure 2(a)). Plasma sP-selectin levels were not significantly lower in the rosiglitazone group than at baseline or in the control group after 6-month treatment (170.2 (119.2 to 251.9) pg/mL versus 182.5 (127.2 to 212.9) pg/mL and 175.2 (119.2 to 251.8) pg/mL, *P* > .05). In addition, plasma levels of sICAM-1 and sP-selectin did not differ from control levels before and after 6-month treatment (Figures 2(a)-2(b)).

**Figure 3:**
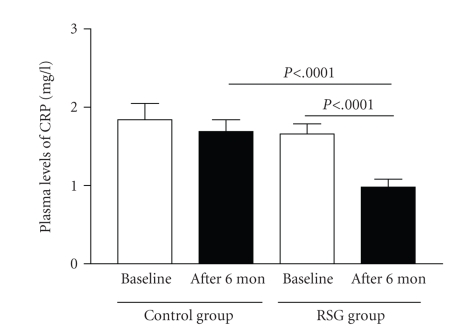
Effect of rosiglitazone treatment on plasma levels of C-reactive protein (CRP) in patients with diabetes and coronary artery disease after percutaneous coronary intervention. Data are means ± SD. Control group, *n* = 56; rosiglitazone (RSG) group, *n* = 60.

Plasma CRP levels in the rosiglitazone group were significantly reduced from 1.66 ± 0.23 to 0.88 ± 0.21 mg/L after 6-month treatment (Figure 3) and were significantly lower than in controls after 6-month treatment (mean 0.88 ± 0.21 versus 1.69 ± 0.25 mg/L, *P* < .0001). CRP levels in the control group did not differ from baseline levels after 6-month follow-up.

### 3.4. Rosiglitazone effect on coronary events during 6-month follow-up

Eleven patients in the rosiglitazone group had coronary events (recurrent unstable angina in 8, percutaneous translational coronary angioplasty in 2, and coronary artery bypass grafting in 1) versus 14 patients in the control group (recurrent unstable angina in 7, percutaneous translational coronary angioplasty in 2, sudden death in 1, and coronary artery bypass grafting in 4) at 6-month follow-up, with no significant difference between the two groups.

## 4. DISCUSSION

Chronic low-grade inflammation is associated with cardiovascular events in patients with CAD or diabetes. Endothelial cell expression of adhesion molecules and the adhesion of leukocytes to the endothelium play a key role in the development of atherosclerotic plaque and plaque instability. Endothelial function may be assessed by measuring plasma levels of endothelial products such as soluble adhesion molecules. Our study demonstrates that 6-month treatment with the PPAR-*γ* rosiglitazone treatment significantly improved metabolic parameters, including insulin resistance in patients with diabetes and CAD. In addition, rosiglitazone treatment significantly decreased the plasma levels of sICAM-1 and inflammatory marker CRP as compared with baseline and control levels.

 Atherosclerosis is characterized by the recruitment of monocytes and lymphocytes to the artery wall. A number of studies have determined the important role of adhesion molecules in atherosclerotic plaque formation [2, 3]. P-selectin is responsible in part for the adhesion of certain leukocytes and platelets to the endothelium. Animal models have also shown the important role of P-selectin in the process of atherogenesis. For example, increased P-selectin expression has been demonstrated in active atherosclerotic plaques. Increased levels of soluble P-selectin in plasma have also been demonstrated in CAD [14]. Several studies have demonstrated plasma levels of CRP positively associated with risk of cardiovascular disease and clinical events. CRP may exert a direct effect in promoting the progression of atherosclerosis and plaque instability [15]. Rosiglitazone significantly reduces levels of markers of endothelial cell activation and CRP in CAD patients without diabetes; potential mechanisms include insulin sensitization and modification of inflammation within the vessel wall [16].

PPAR-*γ* is expressed in most cells of the vascular wall and atherosclerotic lesions [17, 18]. The binding of monocytes to adhesion molecules expressed on the surface of endothelial cells and their infiltration into the subendothelial space may be reduced by PPAR*γ* agonists. The PPAR-*γ* agonist troglitazone inhibits the interaction between leukocyte endothelial cells and decreases the expression of vascular cell adhesion molecule-1 (VCAM-1) and ICAM-1 in activated endothelial cells and reduces monocyte homing to atherosclerotic plaque [9, 10]. In recent years, PPAR-*γ* agonists have been increasingly used to treat patients with type 2 diabetes mellitus. Such treatment is aimed at reducing the incidence of vascular complications, including myocardial infarction and restenosis after PCI. Animal studies have shown that troglitazone prevents neointimal formation after endothelial balloon injury in rats [18]. A recent study showed that pioglitazone suppresses in-stent restenosis by limiting local inflammatory pathways in atherosclerotic rabbits [19]. Clinical studies have shown that pioglitazone reduces neointimal volume after coronary stent implantation in nondiabetic patients [20]. As well, rosiglitazone improves diabetes compensation, significantly reducing VCAM-1 level, and E-selectin concentrations in patients with diabetes [21]. Plasma concentrations of CRP and E-selectin were shown to decrease significantly after rosiglitazone treatment for 3 months in diabetic patients, although concentrations of ICAM-1 and VCAM-1 did not decrease [22]. Recently, we showed that plasma levels of MCP-1 and hyperresponsiveness of low-dose lipopolysaccharide-induced MCP-1 secretion from monocytes were significantly reduced by rosiglitazone treatment in patients with type 2 diabetes and serious vascular disease [15]. Therapy with rosiglitazone decreased the serum levels of MCP-1 in obese type-2 diabetic patients [23]. As well, pioglitazone reduces the levels of ICAM-1 and VCAM-1 in obese patients without diabetes, without affecting soluble E-selectin levels [24].

In the present study, we demonstrated further that rosiglitazone significantly decreased plasma sICAM-1 level in diabetic patients with CAD after PCI. However, plasma levels of sP-selectin were not changed significantly by rosiglitazone treatment. Given that chronic subclinical inflammation is important in atherosclerosis and restenosis after PCI, inhibiting the proinflammatory adhesion molecule sICAM-1 and CRP levels by rosiglitazone, might have potentially beneficial effects in type 2 diabetic patients with CAD.

Patients with insulin resistance also have enhanced risk of atherosclerosis. Chronic inflammation may play an important role in the development of insulin resistance and endothelial dysfunction. PPAR-*γ* agonists may modulate insulin action, which results in change in expression of a number of genes involved in glucose level, lipid metabolism, and inflammation. These changes are associated with the reversal of many components of the insulin resistance syndrome. Hence, in our study, the reduction in inflammatory marker levels may be due to a collective effect of rosiglitazone and better metabolic control. Recently, Ryan et al. [24] reported that pioglitazone treatment improved insulin resistance and endothelial function and reduced arterial stiffness in obese men. Our results, together with previous ones, show that fasting plasma glucose, insulin, and hemoglobin A1c levels, as well as HOMA-IR, are all decreased significantly by 6-month rosiglitazone treatment. Thus, the reversal of the insulin resistance syndrome with rosiglitazone is associated with improved cardiovascular risk factors in patients with diabetes and CAD after PCI.

In conclusion, antidiabetic rosiglitazone therapy may play an important role in protecting endothelial function by normalizing the metabolic disorders of diabetes mellitus and depressing the chronic inflammatory response of the vascular wall, eventually reducing the occurrence of coronary events and restenosis after PCI in type 2 diabetic patients with CAD.

## Figures and Tables

**Table 1:** Baseline clinical characteristics of patients with diabetes and coronary artery disease after percutaneous coronary intervention.

	Control *n* = 56	Rosiglitazone *n* = 60
*Character*		
* *Age, years	63.20 ± 7.50	61.60 ± 7.60
* *Body mass index	25.50 ± 2.70	26.10 ± 2.60
* *Sex (M/F)	45/10	49/11

*Risk factors, no.*		
* *Hyperlipidemia	24	26
* *Hypertension	41	42
* *Smoking	23	28

*Parameters (levels)*		
* *Cholesterol, mmol/L	4.80 ± 0.57	4.85 ± 0.48
* *High-density lipoprotein, mmol/L	1.02 ± 0.15	1.04 ± 0.12
* *Low-density lipoprotein, mmol/L	2.87 ± 0.63	2.93 ± 0.78
* *Triglycerides, mmol/L	2.01 ± 0.70	1.99 ± 0.63
* *Fasting insulin, m*μ*/L	11.40 ± 1.72	13.10 ± 1.84
* *Fasting plasma glucose, (mmol/L)	7.20 ± 1.35	7.10 ± 1.42
* *HbA1c, %	7.23 ± 0.68	7.15 ± 0.72
* *HOMA-IR	3.85 ± 0.38	4.12 ± 0.38
* *CRP, mg/dL	1.84 ± 0.21	1.66 ± 0.23
* *sICAM-1, ng/mL	407.1 (344.3–476.1)	423.5 (327.4–421.9)
* *sP-selectin, ng/mL	172.6 (123.3–223.3)	182.5 (127.2–212.9)

*Medication*		
* *Aspirin	55	60
* *B-blocker	51	53
* *Lipid-lowering drugs	51	58
* *Nitrates	24	23
* *Ca-Antagonists	7	7
* *ACE inhibitors	43	43
* *Other antidiabetic drugs	34	35
* * * *Biguanides	22	24
* * * *Acarbose	8	8
* * * *Sulfonylureas	4	5

HOMA-IR: homeostasis-model-assessment insulin resistance; CRP: C-reactive protein; sICAM-1: soluble intercellular adhesion molecule-1; HbA1c: hemoglobin A1c; sP-selectin: soluble P selectin. Data are means ± SD or medians and ranges.

**Table 2:** Metabolic features of patients before and 6 months after rosiglitazone treatment. Values are mean ± SD.

Metabolic parameters	Control group (*n* = 56)		Rosiglitazone group (*n* = 60)	
	Baseline	after 6 months	Baseline	after 6 months
Total cholesterol, mmol/L	4.80 ± 0.57	4.33 ± 0.51*	4.85 ± 0.48	4.27 ± 0.42^#^
High-density lipoprotein, mmol/L	1.02 ± 0.15	1.26 ± 0.11^#^	1.04 ± 0.12	1.35 ± 0.15^#^
Low-density lipoprotein, mmol/L	2.87 ± 0.63	2.67 ± 0.55	2.93 ± 0.78	2.53 ± 0.73*
Triglycerides, mmol/L	2.01 ± 0.70	1.75 ± 0.61	1.99 ± 0.63	1.62 ± 0.62
Body mass index	25.50 ± 2.7	25.37 ± 2.34	26.17 ± 2.60	26.49 ± 2.11

*P < .05 compared to baseline;

^#^ *P* < .01 compared to baseline.

## References

[1] Ross R (1999). Atherosclerosis—an inflammatory disease. *The New England Journal of Medicine*.

[2] Iiyama K, Hajra L, Iiyama M (1999). Patterns of vascular cell adhesion molecule-1 and intercellular adhesion molecule-1 expression in rabbit and mouse atherosclerotic lesions and at sites predisposed to lesion formation. *Circulation Research*.

[3] Ogawa H, Yasue H, Miyao Y (1999). Plasma soluble intercellular adhesion molecule-1 levels in coronary circulation in patients with unstable angina. *The American Journal of Cardiology*.

[4] O'Malley T, Ludlam CA, Riemermsa RA, Fox KAA (2001). Early increase in levels of soluble inter-cellular adhesion molecule-1 (sICAM-1). Potential risk factor for the acute coronary syndromes. *European Heart Journal*.

[5] Festa A, D'Agostino R, Howard G, Mykkänen L, Tracy RP, Haffner SM (2000). Chronic subclinical inflammation as part of the insulin resistance syndrome: the insulin resistance atherosclerosis study (IRAS). *Circulation*.

[6] Hsueh WA, Jackson S, Law RE (2001). Control of vascular cell proliferation and migration by PPAR-*γ*: a new approach to the macrovascular complications of diabetes. *Diabetes Care*.

[7] Goetze S, Xi X-P, Kawano H (1999). PPAR*γ*-ligands inhibit migration mediated by multiple chemoattractants in vascular smooth muscle cells. *Journal of Cardiovascular Pharmacology*.

[8] Ricote M, Li AC, Willson TM, Kelly CJ, Glass CK (1998). The peroxisome proliferator-activated receptor-*γ* is a negative regulator of macrophage activation. *Nature*.

[9] Jackson SM, Parhami F, Xi X-P (1999). Peroxisome proliferator-activated receptor activators target human endothelial cells to inhibit leukocyte-endothelial cell interaction. *Arteriosclerosis, Thrombosis, and Vascular Biology*.

[10] Pasceri V, Wu HD, Willerson JT, Yeh ETH (2000). Modulation of vascular inflammation in vitro and in vivo by peroxisome proliferator-activated receptor-*γ* activators. *Circulation*.

[11] Zeng X, Dai J, Remick DG, Wang X (2003). Homocysteine mediated expression and secretion of monocyte chemoattractant protein-1 and interleukin-8 in human monocytes. *Circulation Research*.

[12] Wang G, Wei J, Guan Y, Jin N, Mao J, Wang X (2005). Peroxisome proliferator-activated receptor-*γ* agonist rosiglitazone reduces clinical inflammatory responses in type 2 diabetes with coronary artery disease after coronary angioplasty. *Metabolism*.

[13] Yu J, Jin N, Wang G, Zhang F, Mao J, Wang X (2007). Peroxisome proliferator-activated receptor *γ* agonist improves arterial stiffness in patients with type 2 diabetes mellitus and coronary artery disease. *Metabolism*.

[14] Blann AD, Nadar SK, Lip GYH (2003). The adhesion molecule P-selectin and cardiovascular disease. *European Heart Journal*.

[15] Pasceri V, Chang J, Willerson JT, Yeh ETH (2001). Modulation of C-reactive protein-mediated monocyte chemoattractant protein-1 induction in human endothelial cells by anti-atherosclerosis drugs. *Circulation*.

[16] Sidhu JS, Cowan D, Kaski J-C (2003). The effects of rosiglitazone, a peroxisome proliferator-activated receptor-gamma agonist, on markers of endothelial cell activation, C-reactive protein, and fibrinogen levels in non-diabetic coronary artery disease patients. *Journal of the American College of Cardiology*.

[17] Marx N, Schönbeck U, Lazar MA, Libby P, Plutzky J (1998). Peroxisome proliferator-activated receptor gamma activators inhibit gene expression and migration in human vascular smooth muscle cells. *Circulation Research*.

[18] Chen Z, Ishibashi S, Perrey S (2001). Troglitazone inhibits atherosclerosis in apolipoprotein E-knockout mice: pleiotropic effects on CD36 expression and HDL. *Arteriosclerosis, Thrombosis, and Vascular Biology*.

[19] Joner M, Farb A, Cheng Q (2007). Pioglitazone inhibits in-stent restenosis in atherosclerotic rabbits by targeting transforming growth factor-*β* and MCP-1. *Arteriosclerosis, Thrombosis, and Vascular Biology*.

[20] Marx N, Wöhrle J, Nusser T (2005). Pioglitazone reduces neointima volume after coronary stent implantation: a randomized, placebo-controlled, double-blind trial in nondiabetic patients. *Circulation*.

[21] Doležalová R, Haluzík MM, Bošanská L (2007). Effect of PPAR-*γ* agonist treatment on markers of endothelial dysfunction in patients with type 2 diabetes mellitus. *Physiological Research*.

[22] Chu JW, Abbasi F, Lamendola C, McLaughlin T, Reaven GM, Tsao PS (2005). Effect of rosiglitazone treatment on circulating vascular and inflammatory markers in insulin-resistant subjects. *Diabetes & Vascular Disease Research*.

[23] Mohanty P, Aljada A, Ghanim H (2004). Evidence for a potent antiinflammatory effect of rosiglitazone. *The Journal of Clinical Endocrinology & Metabolism*.

[24] Ryan KE, McCance DR, Powell L, McMahon R, Trimble ER (2007). Fenofibrate and pioglitazone improve endothelial function and reduce arterial stiffness in obese glucose tolerant men. *Atherosclerosis*.

